# 

**Published:** 1891-02

**Authors:** 


					﻿FEBRTJJLRY, 1891.
OLD SERIES
VoL XXV
4* 1855 <
NEW SERiUS
Vol. VII.—No. 12.'J

IIGMUM
dUUHM
H. V. M. MILLER, M. D.. LL.D.
VIRGIL 0. HARDON, M. D.
F. W. McRAE, M.|D.
L.	P KENNEDY, A. M.. M. D.
M.	B. HUTCHINS, M D
JAMES P. HARR ISON Al
eXfl"! AKITA PA
SUBSCRIPTION' S2.SO A YKAIL
				

